# Benefits and Challenges of Drug-Coated Balloons in Peripheral Artery Disease: From Molecular Mechanisms to Clinical Practice

**DOI:** 10.3390/ijms25168749

**Published:** 2024-08-11

**Authors:** Dan-Alexandru Tataru, Florin-Leontin Lazar, Horea-Laurentiu Onea, Calin Homorodean, Mihai-Claudiu Ober, Maria Olinic, Mihail Spinu, Dan-Mircea Olinic

**Affiliations:** 1 Medical Clinic No. 1, Internal Medicine Department, University of Medicine and Pharmacy “Iuliu Hatieganu”, 400006 Cluj-Napoca, Romania; tataru.cardio@gmail.com (D.-A.T.); onea.lau91@yahoo.com (H.-L.O.); chomorodean@yahoo.com (C.H.); maria_olinic@yahoo.com (M.O.); spinu_mihai@yahoo.com (M.S.); danolinic@gmail.com (D.-M.O.); 2 Interventional Cardiology Department, Cluj County Emergency Hospital, 400006 Cluj-Napoca, Romania; mihai_ober@yahoo.com; 3 DCB Academy, 20143 Milan, Italy

**Keywords:** drug-coated balloon (DCB), peripheral artery disease (PAD), femoropopliteal (FP), below-the-knee (BTK), in-stent restenosis (ISR), paclitaxel, sirolimus

## Abstract

Multiple clinical trials have reported favorable outcomes after drug-coated balloon therapy for peripheral artery disease in above-the-knee and below-the-knee lesions and in both de novo and in-stent restenosis. However, there are still insufficient data to identify and tackle the risk factors associated with a higher risk of restenosis, which is the primary concern for patients who are treated with an endovascular approach. A modern armamentarium, which includes improved lesion preparation techniques such as plaque modification balloons, mechanical atherectomy, intravascular lithotripsy, and imaging, is crucial for obtaining better long-term clinical outcomes. Moreover, a better understanding of the molecular properties of drug-coated balloons has led to improved devices that could tackle the shortcomings of previous generations. This comprehensive review focuses on drug-coated balloon technology as a tool to treat peripheral artery disease and the effects of the molecular mechanisms involved in preventing vascular restenosis.

## 1. Introduction

There has been a continuous increase in the incidence of peripheral artery disease (PAD) in the last 20 years, despite numerous efforts to improve the management and prevention of this disease. As a result, a report on the global burden of disease published in 2019 mentioned a global prevalence of PAD per 100,000 of 332.32 in males and 621.11 in females in the 40–44 age group, which increased markedly with age [1,2]. These patients represent a real global burden not only due to the significant risk of amputation and, subsequently, poor quality of life, but also due to a higher risk of mortality, myocardial infarction, stroke, and major adverse cardiac events (MACEs) [3,4].

While medical treatment mainly addresses the main symptoms of these patients (claudication, reduced walking distance), revascularization options, especially endovascular treatment, have continuously improved. Atherectomy devices, drug-coated balloons (DCBs), or new stent designs have enhanced long-term vessel patency, which represents the main pitfall of endovascular treatment and occurs in more than 50% of the patients within one year after the procedure [5].

DCBs have shown excellent results in preventing restenosis in patients with coronary in-stent restenosis (ISR). As a result, their use has largely been extended to native coronary and peripheral arteries. The performance of DCBs relies on their ability to homogenously transfer the drug into the vessel wall without the disadvantage of foreign body implantation. Further beneficial effects, such as vasomotion restoration, vessel remodeling, and late lumen enlargement (LLE), are drug- and dose-dependent [6,7]. 

In this manuscript, we revised the most relevant data on the benefits of DCBs for treating lower extremity arterial disease, or PAD, focusing only on atherosclerotic etiology.

## 2. Molecular Basis

DCB technology relies on the capacity of a semi-compliant balloon to efficiently and rapidly release an antiproliferative substance to the vessel wall during balloon inflation [8]. An ideal antiproliferative agent used in DCB angioplasty should possess specific pharmacological properties. The ideal drug should suffer a minimum loss of concentration upon delivery to the target vessel. Additionally, the chosen agent must exhibit high lipophilicity to ensure quick and homogeneous delivery to the target tissue while remaining active in the vessel wall for long-term antiproliferation [9]. Notably, the agent must not exhibit local or systemic toxicity.

There are currently two drugs approved for DCB coating: paclitaxel and sirolimus. Both share a common mechanism of action through the inhibition of cell proliferation and prevention of vascular restenosis. Table 1 summarizes the main characteristics of these drugs. 

Starting from the previously mentioned mechanisms of action, Maga P et al. [10] characterized T cell subsets in forty-five PAD patients undergoing percutaneous angioplasty (PTA) to better address the major limitation of endovascular treatment, represented by significant target lesion revascularization (TLR) rates. In this study, the authors collected two blood samples from the lesion site immediately before and after PTA. They followed acute changes in T cell levels. The reduction observed is likely due to the adhesion of cells to the injured vascular wall, suggesting that immunosenescent, activated T cells have a role in the early vascular injury immune response following PTA in PAD patients. These findings could better enhance the rationale for DCB use, as sirolimus can inhibit the proliferation of immune cells such as T and dendritic cells [11]. At the same time, in animal studies, paclitaxel exerts a complex immune modulatory effect by decreasing regulatory T cells using the anti-CD25 pathway [12].

### 2.1. Paclitaxel

Paclitaxel is a cytostatic chemotherapy drug used systemically in the treatment of several types of carcinoma. It is the first local antiproliferative drug supported by a large body of evidence. Paclitaxel’s primary mechanism of action is based on its ability to bind to the beta subunit of tubulin, thus stabilizing polymerized microtubules, preventing their disassembly, and suppressing mitotic division, resulting in apoptosis [13]. Moreover, it has been demonstrated that paclitaxel has a dose-dependent effect, with lower concentrations of this drug having the ability to trigger programmed cell death [14]. 

An essential advantage of paclitaxel is the increased passive absorption through the cell membrane and prolonged retention in the vessel wall due to its high lipophilic capability [15]. Current paclitaxel-based DCBs used in treating PAD employ a paclitaxel dose ranging from 2.0 to 3.5 µg/mm^2^. In experimental models, paclitaxel caused a dose-dependent increase in tissue necrosis, vascular wall hemorrhage, and delayed healing [16]. However, recent clinical data suggest there is no difference in the efficacy or safety of these devices when comparing low- vs. high-dose paclitaxel coating (primary patency 70.6% vs. 71.4%, *p* = 0.96; all-cause mortality 3.6% vs. 2.2%, *p* = 0.55; TLR 17.3% vs. 13.0%, *p* = 0.31 [17]. 

There are controversial data on the safety associated with paclitaxel devices. Two meta-analyses show a correlation between paclitaxel-based DCBs and an increase in mortality, but loss of follow-up and withdrawal rates were high in these studies [18,19]. On the contrary, large-scale studies [20,21,22] comparing DCBs with standard PTA found no causal relation between paclitaxel use and mortality. Moreover, mortality was lower in the DCB group than in the PTA group [22]. 

The LLE phenomenon seen after DCB angioplasty of coronary arteries [23] led to additional concerns regarding this agent. An exaggerated degenerative effect in the vessel wall, leading to aneurysm formation, was observed.

While aneurysm formation after DES implantation in the femoropopliteal (FP) arteries was estimated at a rate of 8% [24], the incidence of this complication after DCB use is still unknown since it is only documented in case-report papers [25,26]. The mechanisms contributing to pathologic arterial remodeling involve vascular smooth muscle cell migration and proliferation, elastin degradation, collagen deposition, and extracellular matrix calcification. Other possible mechanisms for aneurysm formation include exaggerated wall thinning or plaque regression induced by paclitaxel [27].

Clinical factors that may increase the risk for such remodeling may include the subintimal passage of wires, arterial wall injury from atherectomy, paclitaxel-induced smooth muscle apoptosis, hypersensitivity reactions, inflammation, and inadequate medical therapy [26].

### 2.2. Sirolimus

Sirolimus represents a potent immunosuppressant that primarily acts by binding to the cytosolic protein FKBP12, thus modulating the activity of the mTOR, a protein kinase that regulates cell growth. This, in turn, results in the inhibition of the IL-2-mediated signal transduction, leading to a cell-cycle blockage in the G1-S phase [28]. The drug inhibits smooth muscle cell proliferation and migration, and since it does not induce apoptosis, it has a broader therapeutic window and increased safety margin compared to paclitaxel [29]. Interestingly, it has a negligible effect on endothelial cell function, but in contrast to paclitaxel, it exhibits anti-inflammatory properties. The latter is significant, as neutrophilic leukocyte activation can precipitate adverse cardiovascular events such as restenosis and acute stent thrombosis [30].

Given the above therapeutical advantages and the safety concern regarding paclitaxel device usage, there is currently increased enthusiasm for the use of sirolimus-based DCBs for PAD treatment. However, given its lower lipophilicity, sirolimus represents a more challenging drug in terms of tissue bioavailability. Its efficacy is even more limited in the larger peripheral arteries due to high wash-out and slower penetration rates, reducing its retention within the vessel wall [30]. As a consequence, several absorption enhancers have been studied. The Magic Touch PTA (Concept Medical, Gujarat, India) was the first CE-marketed sirolimus-based DCB to use the Nanolute technology. This DCB achieves 100% sirolimus sub-micron particles through encapsulation in a phospholipid carrier, allowing controlled drug delivery [31]. 

The SELUTION DCB (MedAlliance, Nyon, Switzerland) uses a biodegradable polymer as a carrier that facilitates drug uptake into the vessel wall through micro-reservoirs. Both devices have been proven safe and effective in preclinical and human studies [31,32,33]. Interestingly, data show that sirolimus has a half-life of around 90 days vs. several days to weeks for paclitaxel, thus conferring a theoretical advantage of a longer therapeutical effect [34]. 

Another plausible explanation regarding the therapeutical differences observed between sirolimus and paclitaxel could be related to specific drug behaviors under hypoxic conditions, as encountered in the presence of significant atherosclerotic disease. Hypoxia is a potent trigger for the expression of HIF-1 alpha, which can promote glycolysis and the proliferation of endothelial and smooth muscle cells. Glycolysis may cause vascular inflammation and mitochondrial dysfunction [35]. In in vitro studies on hypoxic cells [36], sirolimus showed superior antiproliferative effects compared to paclitaxel. However, increasing the paclitaxel dose can surpass this limitation, but at the cost of increased toxicity, demonstrating the narrow therapeutic window of this agent.

### 2.3. Balloon Coating/Excipient

Nevertheless, regardless of the active substance, a polymeric coating or a hydrophilic excipient is required to bind to the drug and facilitate dissolution from the balloon to the tissue. Different excipients are currently being used, impacting local drug transfer and concentration [37]. Despite recent advances, a porcine model shows that at least 30% of the loaded paclitaxel is washed out during balloon deployment [38]. While sharing a common mechanism of action, a wide variety of DCBs are currently available (Table 2) that vary in terms of balloon design, polymeric coating, and type of drug used. 

The IN.PACT Admiral DCB (Medtronic, Minneapolis, MN, USA) utilizes urea as an excipient, a non-toxic and ubiquitous endogenous compound. The SeQuent Please DCB (B. Braun, Melsungen, Germany) uses resveratrol, a plant-based natural substance that acts as a matrix builder. Lutonix (CR Bard, Providence, NJ, USA), Ranger (Boston Scientific, Marlborough, MA, USA), and Stellarex DCBs (Philips, Amsterdam, The Netherlands) are all low-dose paclitaxel-based DCBs that use polysorbate/sorbitol, citrate ester, and polyethylene glycol as carriers. The latter may prove advantageous in calcified lesions as it strongly bonds to hydroxyl apatite. The Passeo-18 Lux (Biotronik, Berlin, Germany) employs n-Butyryl tri-n-hexyl citrate, which is found in various medical devices and cosmetics.

**Figure 1:**
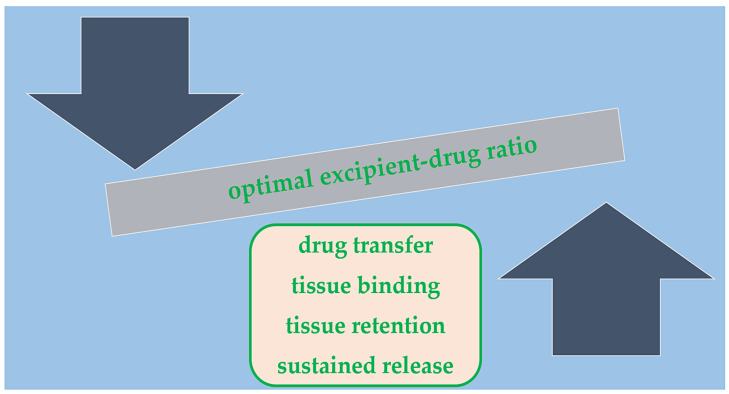
DCB pharmacokinetics properties of drug delivery.

Obtaining a balanced ratio between the lipophilic drug and hydrophilic excipient is crucial, as it can affect DCB pharmacokinetics and efficacy (Figure 1) [39]. Granada et al. [40] tested the difference between a crystalline and amorphous paclitaxel coating formulation (in equal doses). They found similar arterial paclitaxel levels one hour after balloon deployment, but the crystalline form retained higher drug concentrations at both 24 h and 28-day follow-ups. Moreover, the crystalline form produced more important levels of neointimal inhibition and delayed healing. Interestingly, different coating methods can play a role in ensuring an even drug distribution on the balloon surface and preventing downstream wash-out [41].

## 3. Clinical Practice

### 3.1. Lesion Preparation and Imaging

Proper lesion preparation has been demonstrated to contribute to improved peri- and post-procedural clinical outcomes in complex lesions, especially in severe calcifications. Vessel preparation devices include different types of balloons: semi-compliant, non-compliant, scoring, and cutting. Also, mechanical atherectomy includes the following: directional atherectomy (DA), rotational atherectomy (RA), orbital atherectomy (OA), and laser atherectomy (LA). Lastly intravascular lithotripsy (IVL) has gained popularity, and its use is confirmed in severe circumferential calcifications.

#### 3.1.1. Standard, Cutting, and Scoring Balloons

The use of standard balloons (semi-compliant/non-compliant) in preparing lesions and using prolonged inflations (>180 s) increased procedural success rates and long-term patency. Also, it reduces the risk of arterial dissection [42]. Cutting and scoring balloons combine the features of standard balloons with atherotomes or wires mounted on their surface, giving them properties similar to microsurgery blades [43]. Their use allows for controlled plaque incision or dissection, with less barotrauma, lowering the risk of vessel rupture, minimizing plaque shift, decreasing vessel elastic recoil, controlling inflammatory responses, and potentially improving long-term outcomes [43]. 

#### 3.1.2. Orbital Atherectomy

The system consists of a diamond-plated drill placed on a curved shaft with synchronous movement, combining high-speed rotation with sinusoid movement and giving the advantage of removing calcium from all the arterial walls [44].

The Orbital Atherectomy System for the Treatment of Peripheral Vascular Stenosis (OASIS) trial was the first study to investigate the safety and efficacy of OA in PAD. Results showed a high procedural success rate (90.1%) and a low rate of major adverse events (10.4%) at six months [44].

More recently, Zeller et al. [45] randomized 66 patients 1:1 to undergo OA + DCB vs. standard balloon PTA + DCB. The technical success of OA + DCB vs. DCB was 81.8% and 89.2%, respectively. In comparison, the target lesion primary patency rate was numerically higher in the OA + DCB vs. control at the six (88.2% vs. 50.0%, *p* = 0.065) and twelve-month follow-up (88.2% vs. 54.5%, *p* = 0.076), thus suggesting the safety and efficacy of this preparation technique. 

This particular approach was investigated in the CONFIRM registries as well, which included 1109 real-world PAD patients (1544 lesions) suffering from critical limb ischemia (CLI) and treated with OA. It compared the rates of dissection, perforation, slow flow, vessel closure, spasm, embolism, and thrombus formation between different locations: above-the-knee (ATK) lesions vs. below-the-knee (BTK) lesions. OA demonstrated high efficacy regardless of lesion location. BTK lesions were associated with increased rates of perforation, slow flow, and spasm, while ATK lesions had a higher final residual stenosis (*p* = 0.004) and required the use of more adjunctive therapies (1.3% vs. 1.1%; *p* < 0.001) compared to patients with BTK lesions [46].

#### 3.1.3. Rotational Atherectomy

The Rotablator System consists of an elliptical burr plated with nickel and coated with diamond crystals rotating at high speeds, usually ranging from 140,000 to 160,000 rpm [47]. Enough evidence supports the use of rotablation in highly calcific coronary lesions. However, despite early studies, there are still limited data on the outcomes of this technique in PAD patients. 

In 1991, Dorros et al. conducted a study using high-speed rotablation in 38 patients (82 lesions) with a 95% procedural success rate and without major complications [48]. Similarly, Zeller et al. enrolled 172 PAD patients with Rutherford classes 1 to 5. Two hundred and ten lesions (mean length 2.7 cm) were treated with the Pathway PV System [49]. Rotablation appeared safe and efficient. The device success rate was 99%, and major adverse events at 30 days were encountered in 1% of the cases (two preplanned amputations). Clinically driven TLR at 6 and 12 months were 15% (25/172 patients) and 26% (42/162 patients), respectively. The one-year restenosis rate was 38.2% based on duplex ultrasound images [49,50]. 

#### 3.1.4. Directional Atherectomy

DA devices remove plaque by guiding a motorized cutter of the catheter towards the plaque itself and then advancing or rotating the cutter to remove plaque and capture it in its nosecone. These properties make DA ideal for eccentric plaque removal [43]. Several studies reported encouraging results using DA for lesion preparation in PAD patients. 

The Talon registry [51] included 601 patients with 1258 lesions, of which 317 were BTK. The overall procedural success (<50% stenosis) was 97.6%, with a 1-year TLR of 20%. Zeller et al. achieved a high procedural success rate treating 49 BTK lesions with DA. The authors describe high patency rates of 91% after 1 year and 80% after 24 months [52]. 

The DEFINITIVE AR multicenter randomized trial enrolled 102 patients to estimate DA’s effect before DCB angioplasty. Technical success was superior for DA + DCB vs. DCB alone (89.6% vs. 64.2%, *p* = 0.004). The rate of flow-limiting dissections was 2% for DA + DCB vs. 19% for DCB alone, *p* = 0.011. At the one-year follow-up, per cent diameter stenosis was 33.6 ± 17.7% for DA + DCB vs. 36.4 ± 17.6% for DCB, *p* = 0.48. Clinically driven TLR was 7.3% for DA + DCB and 8.0% for DCB, *p* = 0.90. One-year patency, assessed through duplex ultrasound imaging, was numerically higher in the DA + DCB group but without statistical significance (84.6% vs. 81.3%, *p* = 0.78). Freedom from MACEs was similar for both groups (89.3% for DA + DCB and 90.0% for DCB alone, *p* = 0.86) [53]. 

#### 3.1.5. Laser Atherectomy

LA is an excimer laser which uses ultraviolet radiation to vaporize and remove plaque and thrombus from the arterial lumen. This lesion preparation technique was evaluated by Gray et al. in a prospective study, and the authors reported a procedural success rate of 88% with a mean wound area reduction of 89% at six months and a 69% limb salvage rate [54].

Moreover, in a recent study, Ducasse et al. compared three different strategies for treating FP arteries ISR: PTA + DCB vs. excimer LA + PTA + DCB vs. standard PTA [55]. The INTACT trial enrolled 134 subjects with >70% ISR, presenting in Rutherford classes 2–5, and randomized them to one of the three strategies. Procedural and clinical success rates were similar between the three groups (*p* = NS). However, >70% ISR significantly decreased in the LA + PTA + DCB group (30%; *p* = 0.04) compared to PTA + DCB (30.2%; *p* = 0.05) and PTA alone (51.2%). Primary patency was higher after PTA + LA + DCB (log-rank *p* = 0.04) and PTA + DCB (log-rank *p* = 0.02) compared to PTA alone at 12 months (78.7% vs. 70.4% vs. 61.5%) and at 18 months (61.6% and 67.7% vs. 37.3%). No differences in major adverse events between the groups were reported. This trial highlighted the safety and efficacy of excimer LA in combination with DCBs for treating FP lesions [55].

Jiang X et al. [56] recently published the results of a study which enrolled 61 patients who underwent LA combined with DCB in popliteal artery long lesions (7.3 ± 2.8 cm). With a high procedural success rate (95.1%), the mean ankle–brachial index (ABI) was significantly improved from 0.45 ± 0.13 at baseline to 0.90 ± 0.12 after the procedure, 0.88 ± 0.11 at six months, and 0.85 ± 0.12 at 12 months follow-up. Moreover, after a mean follow-up of 28.2 ± 6.1 months, reintervention was performed in only five (8.2%) patients, while the 2-year primary patency rate was 83.5%, thus demonstrating the long-term safety and efficacy of this approach for ‘de novo’ peripheral artery lesions.

#### 3.1.6. Intravascular Lithotripsy

The IVL catheter (IVL^®^; Shockwave Medical, Santa Clara, CA, USA) is a recently developed endovascular technique for calcified lesions with promising results. The ultrasound waves selectively crack intimal and medial calcium, allowing the balloon to prepare the lesion with low pressure. As this plaque modification technique is relatively new, data regarding its safety and long-term patency in PAD patients treated with IVL are still scarce. 

In a small preliminary observational study, Brodmann M et al. achieved 95% procedural success using IVL for BTK lesion preparation and no major adverse events were observed at 30 days [57]. 

Furthermore, the Disrupt PAD III trial, which included 306 patients, found that IVL prior to DCB angioplasty resulted in greater procedural success (65.8% vs. 50.4%; *p* = 0.01), fewer flow-limiting dissections (1.4% vs. 6.8%; *p* = 0.03), and less need for stent placement (4.6% vs. 18.3%; *p* < 0.001) compared to PTA alone prior to DCB deployment [58]. 

A post hoc analysis of this trial, representing the most extensive real-world experience of IVL treatment in heavily calcified common femoral artery lesions, reported encouraging safety results [59]. By core laboratory assessment, no vascular complications were present at the end of the procedure, with only one flow-limiting dissection occurring immediately following IVL treatment. Also, a significant stenosis reduction was reported. Of 177 patients enrolled at 23 sites, DCBs were the final therapy in 68.9% of patients and final stenting in 16.5%.

#### 3.1.7. Imaging Modalities

While various imaging techniques, such as intravascular ultrasound (IVUS) or optical coherence tomography (OCT), have demonstrated improved angiographic and clinical outcomes in coronary artery disease, particularly in complex lesions, there are still limited data on the use of intravascular imaging to enhance clinical outcomes in PAD.

A prospective, multicenter, randomized trial conducted in South Korea compared the outcomes of 237 patients randomized to IVUS-guided (*n* = 119) vs. angiography-guided (*n* = 118) angioplasty for treating FP artery disease with DCBs. The IVUS-guided group showed significantly higher primary patency rates, representing the primary endpoint (83.8% vs. 70.1%, *p* = 0.01). After 12 months, increased freedom from clinically driven TLR (92.4% vs. 83.0%, *p* = 0.02) and sustained clinical improvement (89.1% vs. 76.3%, *p* = 0.01) were in favor of the IVUS-guided group [60]. 

However, these results should be interpreted cautiously, since other studies have reported controversial findings. In a significant study of a group of patients treated with DCBs for FP disease, the authors found only a slight connection between the use of IVUS and a reduced risk of restenosis [61]. Nevertheless, IVUS was able to accurately identify risk factors for restenosis. Horie K et al. found restenosis to be independently associated with chronic total occlusion (*p* < 0.001), circumferential calcification (*p* = 0.023), and a smaller post-procedural minimum lumen area (*p* = 0.036) [62]. 

Recently, OCT has been more commonly used to guide peripheral endovascular treatments and DCB angioplasties. Stavroulakis K et al. [63] included 39 patients treated with an OCT-guided DA + DCB strategy in a retrospective, single-center, single-arm analysis. After a median follow-up of 15 months, the primary patency rate was 93%, and there was no need for TLR. Bailout stenting was required in only 3% of cases (one lesion), and the final angiography did not reveal any flow-limiting dissection. This study suggested that an OCT-guided DA + DCB strategy for FP artery disease may result in sufficient luminal gain and low rates of bailout stenting, with promising 12-month outcomes. However, more extensive trials are needed in order to make specific recommendations.

### 3.2. Clinical Implications

Multiple studies have demonstrated that using an adequate lesion preparation technique led to better angiographic results, improved clinical outcomes, and prolonged vessel patency. 

Tomoi et al. conducted a retrospective study to establish the clinical impact of successful vessel preparation in DCB FP interventions [64]. The study included 268 patients who underwent successful FP intervention using DCBs with a median follow-up of 2.1 years. Successful vessel preparation, defined as <50% residual stenosis and no flow-limiting dissection before DCBs, was achieved in 163 patients (60.8%). Primary patency and freedom from clinically driven TLR were significantly higher in the successful vessel preparation group than in the non-successful group (54.2% vs. 33.0%, *p* < 0.001; 69.9% vs. 57.7%, *p* = 0.047). This study displays the importance of adequate lesion preparation for long-term clinical outcomes.

Imaging is also important, as several studies demonstrated that the IVUS-guided DCB angioplasty of peripheral lesions improved long-term clinical outcomes. In the study conducted by Soga et al. [61], IVUS was used in 73.4% of the cases, which helped select DCBs with diameters larger than the angiographically measured vessel diameter, which may have contributed to the reduction in residual stenosis. Similar results were reported by the more recent IVUS-DCB trial [60].

**Figure 2:**
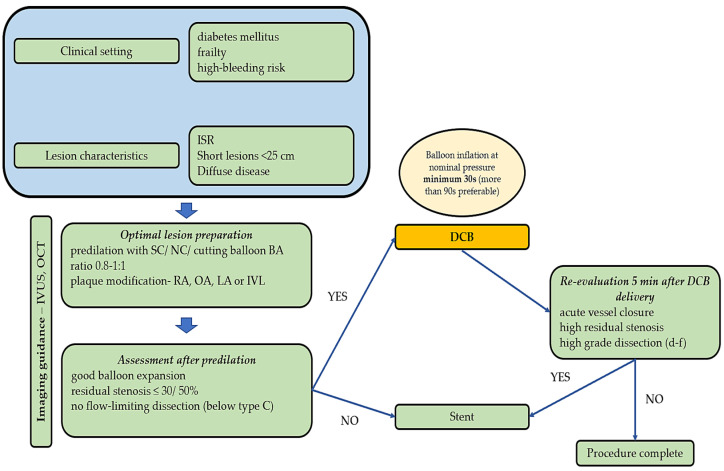
Proposed clinical algorithm for lesion preparation before DCB deployment. (ISR—in-stent restenosis; IVUS—intravascular ultrasound; OCT—optical coherence tomography; SC—semi-compliant; NC—non-compliant; RA—rotational atherectomy; OA—orbital atherectomy; LA—laser atherectomy; IVL—intravascular lithotripsy; DCB—drug-coated balloon).

Figure 2 depicts a clinical algorithm for lesion preparation before DCB deployment and immediate follow-up.

### 3.3. Above-the-Knee Lesions

#### 3.3.1. In-Stent Restenosis

Although the use of DCBs for treating aortoiliac lesions is rare, and very few studies have used this approach, DCBs have become an attractive alternative to stents for treating FP lesions. The rationale is that the implantation of a permanent metal prosthesis in arteries exposed to various external forces from movements has been linked to a higher risk of restenosis (30% of patients at 12 months and up to 50% at 24 months), especially in long lesions or near joints [60,65]. There are currently multiple strategies for treating FP-ISR, including excimer LA, Viabahn PTFE-covered nitinol stents (WL Gore, Flagstaff, AZ, USA), standard PTA, drug-eluting stents (DESs) and DCB angioplasty. The rationale for using paclitaxel-coated balloons for treating ISR is their ability to efficiently reduce the late lumen loss (LLL) in the first 6 to 12 months after angioplasty [66]. 

Several randomized trials have compared DCB use for FP-ISR with other strategies. In the PACUBA trial, 74 patients with symptomatic PAD due to ISR were randomized to either paclitaxel-based DCB angioplasty (*n* = 35) or standard PTA (*n* = 39). At the one-year follow-up, patients treated with the paclitaxel-based DCB had a significantly higher primary patency rate (40.7% vs. 13.4%, *p* = 0.02). However, no difference in clinical parameters such as ABI, improvement in Rutherford class, or clinically driven TLR was reported. 

Similarly, the FAIR trial randomized 119 patients with FP lesions to either standard PTA (*n* = 57) or DCB (*n* = 62) for FP-ISR [67]. The primary endpoint of recurrent ISR assessed using the duplex ultrasound technique at six months was 15.4% (8 of 52) in the DCB group and 44.7% (21 of 47) in the PTA group, *p* = 0.002. 

Interestingly, the mean lesion length included in the FAIR trial was less than half of that reported in the PACUBA trial. This difference might explain the better results reported in the FAIR trial. After one year, freedom from TLR was 90.8% vs. 52.6% (*p* < 0.0001) in favor of the DCB group. Clinical improvement, measured by ≥1 Rutherford class without the need for TLR, was observed in 35 of 45 DCB patients (77.8%) and 23 of 44 PTA patients (52.3%), *p* = 0.015. What is more, no major amputation was needed, and no death was procedure-related [67].

Several other non-randomized trials and studies examined the safety and efficacy of DCBs for FP-ISR. In the DEBATE-ISR Study, 44 consecutive diabetic patients were treated with DCBs for FP-ISR, compared to 42 consecutive diabetic patients treated with standard PTA [68]. At the 3-year follow-up, TLR was 40% in the DCB group vs. 43% in the PTA group (*p* = 0.8), and there were no significant differences in freedom from TLR.

In another small study, Stabile E et al. enrolled 39 consecutive patients who underwent DCB angioplasty of FP-ISR [69]. At one year, the primary endpoint and patency rate were obtained in 92.1% (35 patients), while no patients developed recurrent restenosis assessed using the duplex ultrasound technique.

Considering all these factors, DCBs represent a safe and efficient option for treating FP-ISR, but results from randomized controlled trials are still controversial. However, this strategy improves short-term outcomes, long-term patency, and parameters such as ABI, Rutherford class, or clinically driven TLR for short focal lesions. 

#### 3.3.2. De Novo Lesions

Multiple clinical studies, including randomized controlled trials, demonstrated the safety and effectiveness of DCBs in short symptomatic lesions (6–9 cm) of the FP arteries. In recent years, data from several important trials also have suggested similar benefits for long and complex lesions.

Tepe G et al. [66] conducted a small, multicenter trial including 154 patients with FP lesions randomized to treatment with standard DCBs with paclitaxel, uncoated balloons with paclitaxel dissolved in the contrast medium, or standard balloons (control). The rate of TLR at six months was twenty of fifty-four (37%) in the control group, two of forty-eight (4%) in the group treated with paclitaxel-coated balloons (*p* < 0.001 vs. control), and fifteen of fifty-two (29%) in the group treated with paclitaxel dissolved in the contrast medium (*p* = 0.41 vs. control). At 24 months, the rates increased to twenty-eight of fifty-four (52%), seven of forty-eight (15%), and twenty-one of fifty-two (40%), respectively, thus demonstrating the improved clinical outcomes when using a DCB strategy. 

The PACIFIER trial randomized 85 patients (91 procedures) to either DCB (*n* = 44) or conventional PTA (*n* = 47) treatments for symptomatic FP atherosclerotic disease. The primary endpoint was LLL at six months, and secondary endpoints were binary restenosis and Rutherford class change at six months, TLR and major adverse events at 6 and 12 months. Once again, the results demonstrated the excellent performance of DCBs in de novo FP lesions. DCB angioplasty was associated with significantly lower LLL (−0.01 mm vs. 0.65 mm, *p* = 0.001) and fewer binary restenoses (8.6% vs. 32.4%, *p* = 0.01) at the six-month follow-up [70].

In the LEVANT 2 trial, 476 patients with PAD and angiographically significant FP stenoses were randomized in a 2:1 ratio to angioplasty with a paclitaxel-based DCB or standard PTA. At 12 months, the paclitaxel-based DCB outperformed PTA in terms of primary patency (65.2% vs. 52.6%, *p* = 0.02), as well as the rate of patients free from primary safety events (83.9% vs. 79.0%, *p* = 0.005). However, there were no differences in the rates of death, amputation, thrombosis, or reintervention [71].

The BIOLUX *p*-I trial randomized 60 patients to a paclitaxel-based DCB or standard PTA. The Passeo-18 Lux paclitaxel-coated balloon demonstrated safety and efficacy for FP lesions, outperforming conventional balloon angioplasty in terms of LLL at six months (0.51 ± 0.72 vs. 1.04 ± 1.00 mm, *p* = 0.033) and binary restenosis (11.5% vs. 34.6%, *p* = 0.048). What is more, clinically driven TLR was lower in the paclitaxel-coated balloon group at 12 months (16.0% vs. 52.9%, *p* = 0.020). No major amputations or thrombosis were reported [72]. 

The IN.PACT SFA trial enrolled 331 patients with FP disease randomized in a 2:1 ratio to treatment with DCBs or standard PTA. In this study, DCB treatment was associated with a higher 12-month patency (82.2% vs. 52.4%, *p* < 0.001). DCB angioplasty outperformed PTA in terms of clinical outcomes as well, as TLR was encountered only in 2.4% of patients in the DCB arm compared with 20.6% in the PTA arm (*p* < 0.001) at the 12-month follow-up. Moreover, after an additional year of follow-up, the results reported by the authors demonstrated a durable and superior treatment effect of DCB angioplasty vs. PTA with a significantly higher primary patency (78.9% vs. 50.1%; *p* < 0.001), lower TLR (9.1% vs. 28.3%, *p* < 0.001), and similar functional status improvement with fewer repeat interventions [73]. There were no device- or procedure-related deaths and no major amputations in either group through the 24-month follow-up [74]. 

Two more studies demonstrated the long-term safety and efficacy of DCBs. The 24-month primary patency rate plus the freedom from clinically driven TLR were 71.3% plus 87% [6] and 88.2% plus 92.5% [75], respectively.

These studies compared DCBs with conventional PTA, but DCB angioplasty was compared to other strategies as well. The DRASTICO study enrolled 192 patients undergoing FP intervention, randomly assigned 1:1 to a DCB treatment or drug-eluting stent after successful target lesion pre-dilation. At the 12-month follow-up, no significant differences in terms of TLR (22% vs. 21%, *p* = 0.90) were described between the two groups [76]. 

### 3.4. Below-the-Knee Lesions

#### 3.4.1. De Novo Lesions

It is well known that BTK lesions are associated with poorer long-term patency outcomes and higher restenosis rates [46]. DCBs are a well-established, safe, and efficient therapeutic strategy for FP lesions. However, data for BTK lesions are still lacking. 

The BIOLUX P-II was a first-in-man randomized study investigating the performance of DCB angioplasty in BTK lesions. Seventy-two patients were randomized in a 1:1 ratio to a Passeo-18 Lux DCB (Biotronik AG, Buelach, Switzerland) or a Passeo-18 semi-compliant balloon catheter. The primary safety endpoint (a composite of all-cause mortality, target extremity major amputation, target lesion thrombosis, and target vessel revascularization at 30 days) was 0% in the DCB group vs. 8.3% in the standard PTA group, *p* = 0.239. Patency loss at six months was 17.1% in the DCB group vs. 26.1% in the standard PTA group, *p* = 0.298. Major amputations of the target extremity occurred in 3.3% vs. 5.6%, thus demonstrating the safety and efficacy of DCBs in BTK lesions [77]. 

Furthermore, the IN.PACT DEEP trial, a more significant prospective, multicenter, randomized, controlled trial, enrolled 358 patients with CLI due to BTK lesions, and they were randomized 2:1 to either DCB or conventional PTA treatment. At the 12-month follow-up, the co-primary endpoints represented by TLR and LLL were similar between the two groups: TLR was 9.2% for DCB vs. 13.1% for PTA, *p* = 0.291, and LLL was 0.61 ± 0.78 mm for DCB vs. 0.62 ± 0.78 mm for PTA, *p* = 0.950. Moreover, at six months, the primary safety endpoint represented by a composite of all-cause mortality, major amputation, and clinically driven TLR met the non-inferiority criteria (17.7% vs. 15.8%, *p* = 0.021) [78].

Another critical study, the Lutonix BTK trial, enrolled 442 patients with angiographically significant lesions, randomized 2:1 to DCB or PTA treatments. Freedom from major adverse limb events at 30 days in the DCB arm was non-inferior to the PTA group (99.3% vs. 99.4%, non-inferiority *p* < 0.001). Primary patency and clinically driven TLR were also statistically better for the DCB group than PTA alone at six months [79]. 

Lastly, a systematic review of randomized controlled trials investigating the treatment of BTK arteries with paclitaxel-based DCBs compared with standard PTA for CLI patients reported a significantly reduced TLR in the case of paclitaxel-based DCBs (11.8% crude risk of TLR vs. 25.6% in the control group, *p* = 0.004) [80].

#### 3.4.2. In-Stent Restenosis

Several studies have reported good mid-term outcomes for DES treatment in BTK lesions. In a meta-analysis of seven randomized trials enrolling 801 patients (392 DES-treated patients and 409 control patients), with a median follow-up of 12 months, DES improved rates of primary patency (Odds Ratio (OR) 3.49, *p* < 0.00001), freedom from TLR (OR 2.19, *p* = 0.003), major amputation (OR 0.56, *p* = 0.049), and Rutherford class (OR 1.62, *p* = 0.046), but not mortality (OR 1.05, *p* = 0.912) compared to control [81]. However, DES technology has several limitations, particularly the long-term risk of restenosis, and their use is still controversial for BTK lesions. 

Moreover, the use of DCBs or scaffolds has yet to be approved in the USA, except for the Tack device (Philips, Amsterdam, The Netherlands) used in flow-limiting dissections [82]. For BTK ISR, treatment with DCBs has emerged as a promising tool, but data still need to be provided regarding long-term clinical results, as stent placement in BTK lesions is still off-label.

### 3.5. Future Perspectives

A new randomized trial will compare DCB angioplasty to open vascular surgery. The primary endpoint will be vessel patency at 12 months. The secondary objectives include the TLR rate, limb salvage rate, MACEs, quality of life change, and Rutherford class [83]. The results of this study are of great interest, as DCB technology has been compared to endovascular techniques in the past. However, data regarding the comparison of DCB angioplasty with open surgery are still lacking.

Moreover, data remain contradictory when open surgery is compared to any endovascular approach. The BASIL trial randomized 452 patients with severe limb ischemia to either a surgery-first (*n* = 228) or angioplasty-first (*n* = 224) strategy. It reported no significant differences in amputation-free survival between the two strategies after a six-month follow-up [84]. The BEST-CLI study suggested that open surgery has better results than PTA if the patients receive a venous graft bypass [85]. However, as direct comparisons between DCB angioplasty and surgical revascularization are lacking, the results of future studies are needed. 

The interest in using DCB angioplasty in PAD patients has led to the development of newer technologies which are currently being tested. A multicenter Chinese trial (NCT05415995) will compare a novel paclitaxel-based DCB (Zylox-Tonbridge, Zhuhai, China) with a similar DCB produced by Acotec (Beijing, China) in 202 patients with BTK disease. Immediate post-procedural safety and efficacy and a 6-month primary patency at the target lesion, change of ABI, and TLR will be evaluated. The Acoart SCB SFA trial (NCT04982367) will evaluate the safety and efficacy of a sirolimus-based DCB produced by Acotec compared to a paclitaxel-based DCB in patients with FP lesions. The study’s primary endpoint is the 12-month vessel patency rate. Another clinical study (NCT04525794) will assess the safety and clinical performance of a sirolimus-based DCB (Biotronik, Berlin, Germany) in FP lesions in terms of LLL, major adverse events, TLR, amputation rate, binary restenosis, and change in ABI or quality of life.

## 4. Conclusions

DCBs show promise as an alternative to standard PTA for most peripheral artery lesions, regardless of complexity or location. Multiple studies have demonstrated improved patency rates in the mid- and long-term with this technology. The favorable results may be attributed to the ability of paclitaxel, the primary drug used in these balloon catheters, to penetrate the vessel wall and promote LLE and vessel healing. DCB or DES angioplasty is still considered off-label in BTK lesions. On the other hand, some data suggest that DCBs improve the patency rate and reduce the need for stenting, especially in BTK lesions. However, more data on the long-term clinical outcomes of these devices are needed, and there is an urgent need for studies to support their use in BTK lesions.

## Figures and Tables

**Table 1:** The main characteristics of paclitaxel and sirolimus.

	Paclitaxel	Sirolimus
**Biochemical Properties**		
Cytostatic	YES	YES
Cytotoxic	YES	NO
Apoptotic	YES	NO
Antiproliferative	YES	YES
Lipophilic	YES	NO
Suppress neutrophilic leukocyte activation	NO	YES
Highly effective during hypoxic	NO	YES
Effects in normoxic conditions	YES	YES
Broader therapeutic window	NO	YES
**Drug-Coated Balloon Characteristics**		
Similar coating method	YES	NO
Late vessel remodeling	YES	NO

**Table 2:** Types of DCBs used for coronary and peripheral interventions.

Product	Company	Drug Dose (µg/mm^2^)	Excipient
PACLITAXEL-DCB			
IN.PACT Admiral	Medtronic	3.5	Urea
Lutonix	CR Bard	2.0	Polysorbate and sorbitol
Stellarex	Philips	2.0	Polyethylene glycol
SeQuent Please	B. Braun	3.0	Resveratrol
LEGFLOW	Cardionovum (Bonn, Germany)	3.0	Shelloic acid
Ranger	Boston Scientific	2.0	Citrate ester
Passeo-18 Lux	Biotronik	3.0	Butyryl-tri-hexyl citrate
Luminor	iVascular (Barcelona, Spain)	3.0	Organic ester
Surveil	SurModics (Eden Prairie, MN, USA)	3.2	Proprietary photo-link
SIROLIMUS-DCB			
Magic Touch PTA	Concept Medical	1.27	Phospholipid-based
SELUTION	MedAlliance	1.0	PLGA and phospholipid micro reservoir

DCB: drug-coated balloon; PLGA: poly lactic-co-glycolic acid.

## Data Availability

Not applicable.
